# Reappraisal of VEGF in the Pathogenesis of Kawasaki Disease

**DOI:** 10.3390/children9091343

**Published:** 2022-09-01

**Authors:** Chun-Yu Chen, Shih-Hui Huang, Kuang-Jen Chien, Tsung-Jen Lai, Wei-Hsiang Chang, Kai-Sheng Hsieh, Ken-Pen Weng

**Affiliations:** 1 Congenital Structural Heart Disease Center, Department of Pediatrics, Kaohsiung Veterans General Hospital, Kaohsiung 813414, Taiwan; 2 Department of Pediatrics, Chi Mei Medical Center, Tainan 71004, Taiwan; 3 Department of Nursing, Fooyin University, Kaohsiung 83102, Taiwan; 4 Division of Clinical Laboratory, Department of Pathology and Laboratory Medicine, Kaohsiung Veterans General Hospital, Kaohsiung 813414, Taiwan; 5 Department of Food Safety/Hygiene and Risk Management, Medical College, National Cheng Kung University, Tainan 701401, Taiwan; 6 Research Center of Environmental Trace Toxic Substances, National Cheng Kung University, Tainan 701401, Taiwan; 7 Department of Pediatrics, China Medical University Children’s Hospital, Taichung 40447, Taiwan; 8 School of Medicine, National Yang Ming Chiao Tung University, Taipei 11221, Taiwan

**Keywords:** coronary artery lesions, Kawasaki disease, vascular endothelial growth factor

## Abstract

Vascular endothelial growth factor (VEGF) is an important factor in mediating the inflammation of Kawasaki disease (KD). The literature regarding the relationship between VEGF and KD is sparse. The purpose of this study was to investigate the correlation of VEGF and KD. In a prospective study of 42 Taiwanese KD patients (18.9 ± 12.2 months, M/F 22/20) treated with intravenous immunoglobulin (IVIG), a series of VEGF levels was measured from the acute to convalescent phases. KD patients were classified into two subgroups with (*n* =20) and without (*n* = 22) acute coronary artery lesions (CALs). Control samples were obtained from 30 febrile controls (19.1 ± 13.7 months, M/F 13/17). In KD patients, VEGF levels in the pre-IVIG acute phase were significantly higher than those in the subacute and convalescent phases (both *p* < 0.001). In KD patients with CALs, VEGF levels significantly increased immediately in the post-IVIG phase (*p* = 0.039), and then significantly decreased in the subacute phase (*p* = 0.002). KD patients with acute CALs had higher median VEGF levels than those without acute CALs from acute to convalescent phases. In the subacute phase, KD patients with acute CALs had significantly higher VEGF levels (*p* = 0.022) than those without acute CALs. Our data show that VEGF did not decrease after IVIG treatment, and increased significantly after IVIG treatment in KD patients with acute CALs in acute phase. VEGF might be related to the complications of CALs in KD patients.

## 1. Introduction

Kawasaki disease (KD) is a systemic vasculitis, predominantly affecting children less than 5 years of age [1,2]. KD patients are susceptible to developing complications through formation of coronary artery lesions (CALs) if diagnosis and treatment are delayed. Treatment with intravenous immunoglobulin (IVIG) can decrease the incidence of CALs from 15–20% to <5%, but 8–38% of IVIG-resistant KD patients are at high risk for CALs [3,4]. It is an important issue to identify high-risk factors for CALs in the early stage of KD and start additional anti-inflammatory therapy.

Strong immune reactivation and secretion of cytokines have represented the pathogenesis of KD [4,5]. Vascular leakage and tissue edema are key features during the initial phase of KD [4,6]. The degree of edema serves as an important predictor of CALs in KD [7,8]. Vascular endothelial growth factor (VEGF) is related to angiogenesis and lymphangiogenesis [9]. Elevated VEGF levels have been reported to be associated with the risk of developing CALs in KD patients [10,11,12]. Maeno et al. reported that serum VEGF levels in KD patients with CALs increased from a low level in the acute phase to a very high level in the subacute phase, but patients without CALs had high VEGF levels only in the acute phase of KD [10]. Ohno et al.’ study showed that VEGF level was a major risk factor for the development of CALs in KD patients [11]. The findings in the Takeshita et al. study demonstrated that KD patients with a high VEGF/endostatin ratio were at high risk of CALs [12]. The murine model in the Lin et al. study showed that local VEGF-A and its signaling pathway are associated with the occurrence of CALs [13]. Despite the evidence for supporting the correlation of VEGF with CALs in KD, further research is needed to elucidate the effect of VEGF on the outcome of KD patients. 

The purpose of this study is to evaluate the correlations of VEGF with clinical features in different phases of KD, especially formation of CALs in KD children.

## 2. Materials and Methods

### 2.1. Subject Enrollment

This prospective study consisted of 42 KD children, who received IVIG (2 g/kg) therapy in the acute phase at the Department of Pediatrics, Kaohsiung Veterans General Hospital (KVGH), Taiwan. Medical chart review was conducted for the following data: age, sex, symptoms, times of IVIG treatment, complications, and laboratory data as follows: white blood cell (WBC), platelet count, hemoglobin level, alanine aminotransferase (ALT) level, aspartate aminotransferase (AST) level, lipid profile, and C-reactive protein (CRP) level. IVIG resistance is defined as persistent fever for 3 days after initial IVIG treatment. KD patients received two-dimensional echocardiography at the time of diagnosis and again at weeks 2, 4, 8, and 12 after treatment. Acute CALs based on Z score are defined as Z score ≥ 2 within 2 weeks of illness. KD patients were divided into two subgroups with/without acute CALs [4,14]. The control group comprised 30 age- and sex-matched febrile controls with a clinically viral syndrome and without individual history of KD, autoimmune, allergic, or inflammation-associated diseases. This research was performed after the approval of the Institutional Review Board of KVGH (IRB number: VGHKS10-CT9-04). All guardians signed the informed consent form.

### 2.2. Blood Sample Collection

Blood (about 3 mL) was obtained from KD patients in the pre-IVIG acute phase (5.2 ± 1.6 days from initial fever), post-IVIG acute phase (7.9 ± 2.3 days from initial fever), subacute phase (39.2 ± 18.6 days from initial fever), and convalescent phase (182.0 ± 92.8 days from initial fever). Blood (about 3 mL) was obtained once in the acute phase (4.8 ± 1.9 days from initial fever) from febrile controls with a clinically viral syndrome. Blood samples were processed in aliquots of 1 mL of serum. Serum samples were then frozen at −80 °C until analysis of VEGF.

### 2.3. Determination of Serum VEGF

Serum VEGF concentration was measured by the sandwich enzyme-linked immunosorbent assay kit for human VEGF (Quantikine human VEGF; R&D System, Minneapolis, MN, USA). The assay was performed according to the manufacturer’s instructions. The reaction mixture was quantified using the Bio-Plex protein array reader. The VEGF levels were automatically calculated by Bio-Plex Manager software using the appropriate standard curve. The detection limit of VEGF was 31.2 pg/mL. 

### 2.4. Statistical Analysis

Data were expressed as mean ± standard deviation or median (quartiles). Student t test, Mann–Whitney U test, chi-square test, and Wilcoxon signed-rank test were used to determine differences in age, gender, and laboratory data between KD patients with/without acute CALs, and between KD patients and febrile controls. The correlation between VEGF and other laboratory data in KD patients was analyzed by Spearman’s rank correlation test. The receiver operating characteristic (ROC) curve was used to calculate the cutoff values of VEGF to predict KD with acute CALs. The best possible cutoff value was indicated by the highest Youden’s index. A *p* value of less than 0.05 was considered statistically significant. Statistical analyses were performed using SPSS version 22.0 (SPSS Inc., Chicago, IL, USA).

## 3. Results

Forty-two KD patients (M/F 22/20; mean age, 18.9 ± 12.2 months) and thirty febrile controls (M/F 13/17; mean age 19.1 ± 13.7 months) were enrolled in this study. The numbers of KD patients with/without acute CALs were 20 and 22, respectively. No KD patients had IVIG resistance (Table 1). Z scores of the coronary artery were 2.7 ± 0.5 in KD patients with acute CALs and 1.3 ± 0.6 in those without acute CALs. There were no KD patients with myocarditis with systolic dysfunction, arrhythmias, or pericarditis with pericardial effusion.

**Figure 1:**
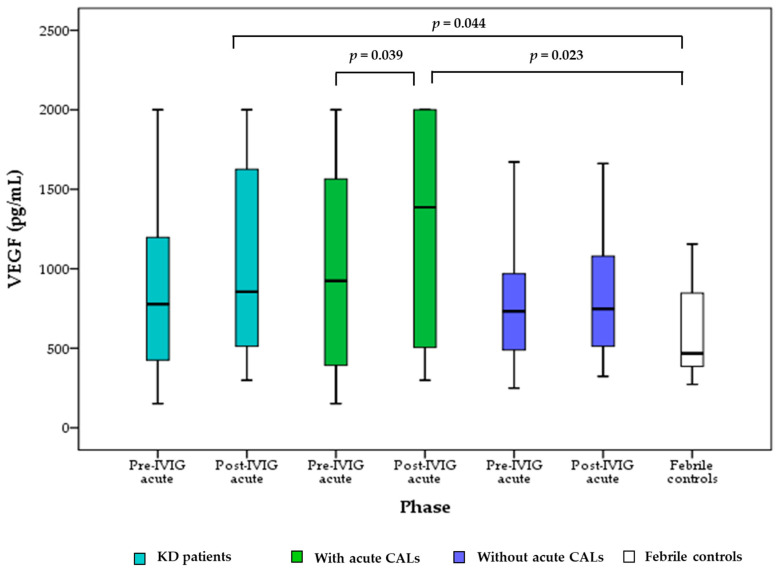
Comparisons of vascular endothelial growth factor (VEGF) levels between KD patients with/without acute CALs in acute phase and febrile controls. Central box, values from the lower to upper quartile (25th–75th percentile). In the box plots, the middle line represents the median. The *p*-value was estimated by the Wilcoxon sign-rank test in KD patients in different phases. The *p*-value was estimated by the Mann–Whitney U test between KD patients and febrile controls.

As shown in Figure 1, KD patients in the post-IVIG acute phase had significantly higher VEGF levels than febrile controls (*p* = 0.044). In KD patients with acute CALs, the VEGF levels significantly increased (*p* = 0.039) from the pre-IVIG acute phase to the post-IVIG acute phase. VEGF levels in KD patients with acute CALs were also significantly higher (*p* = 0.023) in the post-IVIG acute phase compared to those in the febrile controls. In KD patients without acute CALs, VEGF levels did not significantly increase from the pre-IVIG acute phase to the post-IVIG acute phase. KD patients without acute CALs did not have significantly higher VEGF levels in the acute phase than the febrile controls.

In KD patients, VEGF levels in the pre-IVIG acute phase were significantly higher than those in the subacute and convalescent phases (899.5 ± 549.1 vs. 508.9 ± 389.4 and 449.5 ± 362.8 pg/mL, both *p* < 0.001). In KD patients with acute CALs, VEGF levels significantly decreased (1241.8 ± 689.3 vs. 654.7 ± 443.3 pg/mL, *p* = 0.002) from the post-IVIG acute phase to the subacute phase. In KD patients without acute CALs, VEGF levels in the post-IVIG acute stage were also significantly higher (861.0 ± 483.8 vs. 376.4 ± 282.1 pg/mL, *p* < 0.001) than those in the subacute phase. 

**Figure 2:**
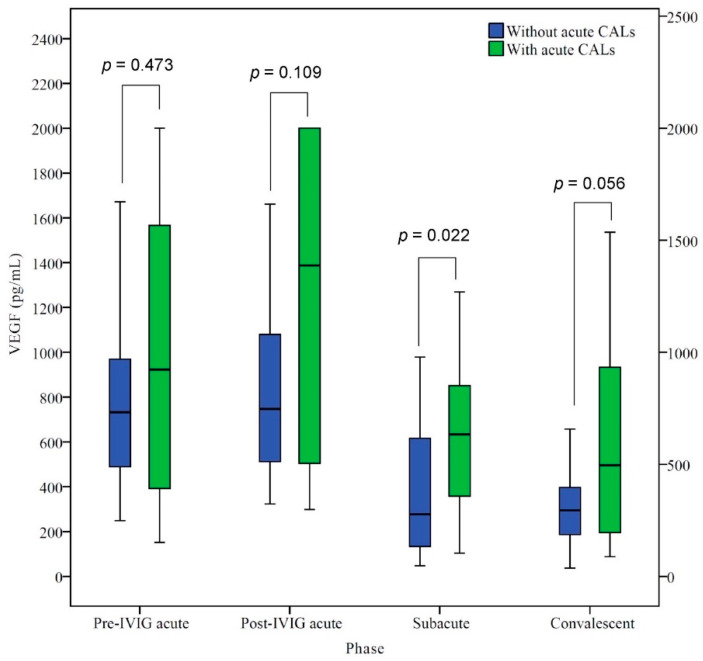
Comparisons of vascular endothelial growth factor (VEGF) levels between KD patients with/without acute CALs. Central box, values from the lower to upper quartile (25th–75th percentile). In the box plots, the middle line represents the median. The *p*-value was estimated by the Mann–Whitney U test.

The difference in VEGF levels at various phases between KD patients with/without acute CALs is shown in Figure 2. KD patients with acute CALs had higher median VEGF levels than those without acute CALs in the various phases. VEGF ratios (with acute CALs/without acute CALs) from the acute to the convalescent phase were as follows: 1.2, 1.4, 1.7., and 1.8, respectively. However, there was no significant difference in VEGF levels in the various phases except the subacute phase. In the subacute phase, KD patients with acute CALs had significantly higher VEGF levels (*p* = 0.022) than those without CALs.

Table 2 shows Spearman correlation coefficients between VEGF levels and the laboratory data at the acute pre-IVIG and post-IVIG phases in all KD patients. In the acute pre-IVIG stage, VEGF was significantly negatively related to albumin (*p* = 0.039), and significantly positively related to platelet (*p* = 0.021) as well as C-reactive protein (CRP) (*p* = 0.021). In the acute post-IVIG phase, VEGF was still significantly positively related to platelet (*p* = 0.036) as well as CRP (*p* = 0.003).

**Figure 3:**
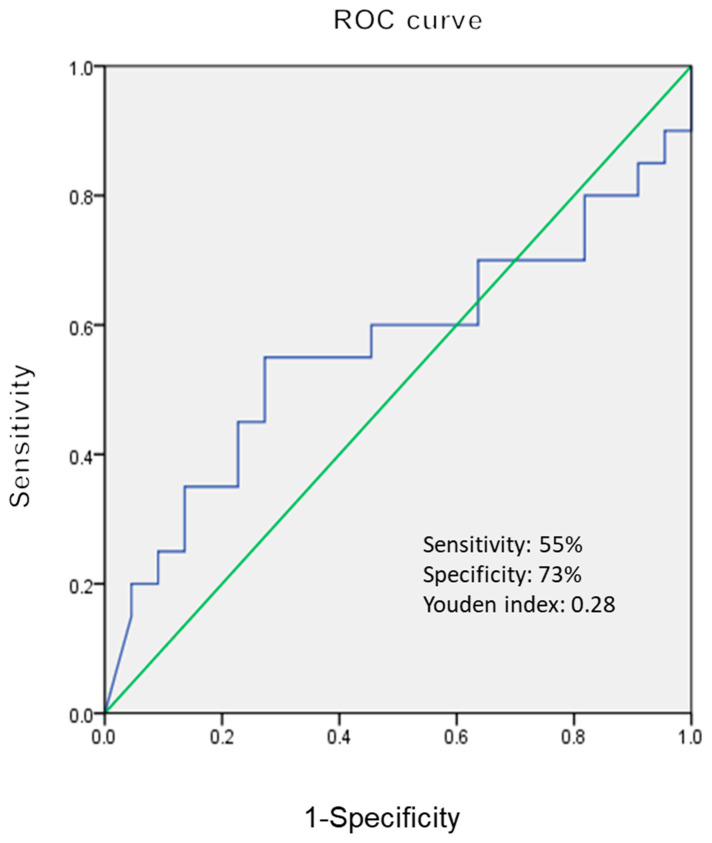
ROC curve for differentiating KD patients with/without acute CALs in the pre-IVIG acute phase at the cutoff value of VEGF 902.4 pg/mL.

Figure 3 shows the best cutoff value of VEGF 902.4 pg/mL to differentiate KD patients with/without acute CALs in the pre-IVIG acute phase.

**Figure 4:**
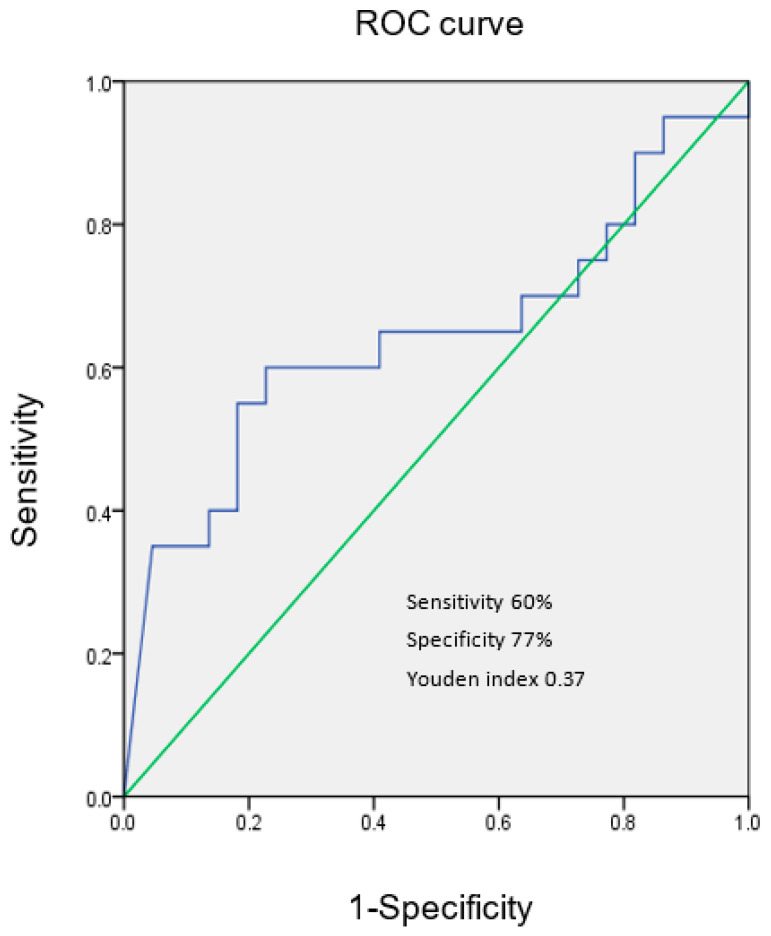
ROC curve for differentiating KD patients with/without acute CALs in the post-IVIG acute phase at the cut-off value of VEGF 1106.7 pg/mL.

Figure 4 shows the best cutoff value of VEGF 1106.7 pg/mL to differentiate KD patients with/without acute CALs in the post-IVIG acute phase.

## 4. Discussion

This study demonstrated that VEGF level in KD patients with acute CALs increased significantly after IVIG treatment in the acute stage. The significant positive correlation of VEGF with CRP as well as platelet in KD patients was found in both pre-IVIG and post-IVIG acute phases. In contrast, there was a negative correlation of VEGF with albumin only in the acute pre-IVIG phase. Compatible with previous studies [6,7,8,9,10,11,12,13], our results confirmed the involvement of VEGF in the systemic vasculitis of KD. The results in this series further imply that VEGF might play an important role in the pathogenesis of KD patients with acute CALs.

In this series, there was a negative correlation of VEGF with albumin in the acute pre-IVIG phase in KD patients. The literature regarding the effect of VEGF on the clinical features of KD is inconsistent. Maeno et al.’s results showed that serum VEGF levels did not correlate with the inflammatory markers and clinical symptoms in KD patients (*n* = 20) [10]. The increased VEGF levels in acute KD patients (*n* = 30) were independent of IVIG therapy and albumin as well as CRP levels in Terai et al.’s study [15]. In contrast, elevation of serum VEGF levels was negatively significantly correlated with low serum albumin in acute KD patients (*n* = 220, r = −0.53, *p* < 0.001) in Yasukawa et al.’s study [16]. Hung et al. also reported that VEGF levels were inversely correlated with albumin levels in acute KD patients (*n* = 25) [9]. Zhou et al. reported that VEGF and platelet are the important risk factors for KD patients with CALs complications (*n* = 60) [17]. All Yasukawa et al.’s [16], Hung et al.’s [9], and our findings might partially support the role of vascular leakage as a key feature of KD pathophysiology. We further found the significant positive correlation of VEGF with CRP as well as platelet in KD patients in both pre-IVIG and post-IVIG acute phases. CRP is a powerful inflammatory biomarker of acute KD, and has been used for predicting IVIG resistance in some scoring systems [18,19,20,21]. In this series, VEGF did not decrease after IVIG treatment, and increased significantly after IVIG treatment in KD patients with acute CALs. Our findings might suggest that high VEGF level is another risk factor for CALs other than IVIG resistance. Platelets are the major source of VEGF in the blood, and platelet VEGF levels in KD patients (*n* = 80) were reported to be significantly correlated with CALs in Ueno et al.’s study [22]. The previous findings [17,22] may partially explain the significant positive correlation of VEGF and platelet in our KD patients in the acute stage. Further studies are required to elucidate the interaction of VEGF, CRP, and platelet in the pathogenesis of KD.

In this series, KD patients with acute CALs had higher median VEGF levels than those without acute CALs in the various phases, but there is a significant difference in VEGF levels between the two groups only in the subacute phase. The ROC curve analysis further showed that the cutoff values of VEGF (902.4 and 1106.7 pg/mL, respectively) in the acute phase could differentiate KD with/without acute CALs with modest sensitivity and specificity. We speculate that the persistent high VEGF levels would be a prognostic marker for CALs complications and related outcomes in KD patients. Our results are generally in agreement with the previous studies [10,11,12]. The effect of VEGF on the CALs in KD patients has been demonstrated in both the immunohistochemical and animal studies. Suzuki et al.’s immunohistochemical study showed that extensive expression of VEGF was observed in the smooth muscle cells of the thickened intima at stenotic sites and at recanalized vessels in KD patients [23]. The immunohistochemical examination in Suzuki el al.’s research showed that VEGF was expressed in the intimal smooth muscle cells of the KD, but not the normal coronary artery without a history of KD [24]. Breunis et al. also reported that immunohistochemistry demonstrated VEGF expression in the coronary artery wall in autopsy tissue [25]. The murine model in Lin et al.’s study showed that local VEGF-A and its signaling pathway are associated with the development of CALs [13]. The rabbit model in An et al.’s study indicated that the phosphatase and tensin homo-log/phosphoinositide 3-kinase/VEGF pathway is important in the vascular injury in KD [26]. It is still a controversial issue to treat IVIG-resistant KD with an additional agent. Miura et al.’s study showed that both additional IVIG therapy and MTP pulse in IVIG-resistant KD patients (*n* = 15) suppressed TNF-α, but not VEGF [27]. Hirono et al.’s research showed that serum levels of proinflammatory cytokines decreased dramatically after infliximab treatment, but VEGF level was not suppressed in IVIG-resistant KD patients (*n* = 11) [28]. Recently, Su et al. reported that IL-35 levels in KD patients (*n* = 90) were negatively associated with VEGF levels, and suggested IL-35 may have the potential effect of preventing KD patients from developing CALs [29]. The potential agent against VEGF merits further investigation in terms of decreasing the risk of CALs in IVIG-resistant KD patients.

There are some limitations in this study. First, this is an observational study with a small number of patients in a single center. However, serial measurement of VEGF levels in four different phases increases the statistical power in this series. Second, the control group consisted of febrile subjects without definite viral diagnosis that had VEGF measurement only in the acute stage. VEGF is an important biomarker in various viral infections, including COVID-19 infection [30,31]. The role of VEGF and associated biomarkers will require in vivo and in vitro studies for clinical utility in the future. Third, the relationship between VEGF and IVIG resistance cannot be analyzed because of the lack of IVIG-resistant KD patients in this series. Fourth, the definition of CALs based on Z score may not reflect the real situation of coronary arteritis and myocarditis in KD patients. All KD patients in this series were IVIG-sensitive and had no echocardiographic findings of myocarditis with systolic dysfunction, arrhythmias, or pericarditis with pericardial effusion. Therefore, the statistical deviation can be decreased when comparing KD patients with/without acute CALs. Further multiple-center studies with large numbers of subjects are suggested.

## 5. Conclusions

Our data show that VEGF did not decrease after IVIG treatment, and increased significantly after IVIG treatment in KD patients with acute CALs in the acute phase. VEGF might be related to the complications of CALs in KD patients.

## Figures and Tables

**Table 1:** Demographic data of subjects.

Factors\Category	KD Patients (*n* = 42)	Febrile Controls (*n* = 30)	*p* Value
	*n* (%)	*n* (%)	
Sex			
Male	22 (52.4)	13 (43.3)	0.537
Female	20 (47.6)	17 (56.7)	
Age (months)	18.9 ± 12.2	19.1 ± 13.7	0.835
Acute CALs			
Yes	20 (47.6)		
No	22 (52.4)		
IVIG resistance			
Yes	0 (0%)		
No	42 (100%)		

KD: Kawasaki disease; CALs: coronary artery lesions; IVIG: intravenous immunoglobulin.

**Table 2:** The correlation between VEGF and laboratory data of KD patients in the acute pre-IVIG and post-IVIG phases.

Variables	Pre-IVIG Acute Phase (*n* = 42)		Post-IVIG Acute Phase (*n* = 42)	
	Correlation Coefficient	*p* Value	Correlation Coefficient	*p* Value
WBC	0.024	0.881	0.008	0.960
PMN	0.188	0.234	0.201	0.203
Lymphocyte	−0.063	0.692	−0.200	0.204
Monocyte	0.030	0.849	−0.026	0.869
Eosinophil	−0.008	0.960	0.201	0.201
Albumin	**−0.320**	**0.039**	−0.041	0.804
Hemoglobin	−0.050	0.754	0.092	0.561
Platelet	**0.356**	**0.021**	**0.325**	**0.036**
AST	0.028	0.861	−0.095	0.549
ALT	−0.063	0.691	−0.069	0.663
CRP	**0.311**	**0.045**	**0.443**	**0.003**
TG	−0.073	0.647	0.280	0.072
TC	−0.055	0.730	0.005	0.976
HDL	−0.207	0.189	−0.110	0.490
TC/HDL ratio	0.142	0.368	0.086	0.588
LDL	−0.031	0.844	0.019	0.906
D-dimer	0.036	0.823	0.139	0.381

KD: Kawasaki disease; VEGF: vascular endothelial growth factor; WBC: white blood cell; PMN: polymorphonuclear; AST: aspartate aminotransferase; ALT: alanine aminotransferase; CRP: C-reactive protein; TG: triglyceride; TC: total cholesterol; HDL: high-density lipoprotein; LDL: low-density lipoprotein.
